# Effect of combined treatment with alendronate and calcitriol on femoral neck strength in osteopenic rats

**DOI:** 10.1186/1749-799X-3-51

**Published:** 2008-12-17

**Authors:** Yoshinari Nakamura, Masatoshi Naito, Kazuo Hayashi, Abbas Fotovati, Samah Abu-Ali

**Affiliations:** 1 Department of Orthopaedic Surgery, Fukuoka University School of Medicine, Fukuoka, Japan; 2 Rheumatology and Arthritis Center Fukuoka Wajiro Hospital 2-2-75, Wajirooka, Higashi-ku, Fukuoka, Japan

## Abstract

**Background:**

Hip fracture is associated with pronounced morbidity and excess mortality in elderly women with postmenopausal osteoporosis. Many drugs have been developed to treat osteoporosis and to reduce the risk of osteoporotic fractures. We investigated the effects of combined alendronate and vitamin D_3 _treatment on bone mass and fracture load at the femoral neck in ovariectomized (OVX) rats, and evaluated the relationship between bone mass parameters and femoral neck strength.

**Methods:**

Thirty 12-week-old female rats underwent either a sham-operation (n = 6) or OVX (n = 24). Twenty weeks later, OVX rats were further divided into four groups and received daily doses of either saline alone, 0.1 mg/kg alendronate, 0.1 μg/kg calcitriol, or a combination of both two drugs by continuous infusion via Alzet mini-osmotic pumps. The sham-control group received saline alone. After 12 weeks of treatment, femoral necks were examined using peripheral quantitative computed tomography (pQCT) densitometry and mechanical testing.

**Results:**

Saline-treated OVX rats showed significant decreases in total bone mineral content (BMC) (by 28.1%), total bone mineral density (BMD) (by 9.5%), cortical BMC (by 26.3%), cancellous BMC (by 66.3%), cancellous BMD (by 29.0%) and total cross-sectional bone area (by 30.4%) compared with the sham-control group. The combined alendronate and calcitriol treatments improved bone loss owing to estrogen deficiency. On mechanical testing, although OVX significantly reduced bone strength of the femoral neck (by 29.3%) compared with the sham-control group, only the combined treatment significantly improved the fracture load at the femoral neck in OVX rats to the level of the sham-controls. The correlation of total BMC to fracture load was significant, but that of total BMD was not.

**Conclusion:**

Our results showed that the combined treatment with alendronate and calcitriol significantly improved bone fragility of the femoral neck in OVX osteopenic rats.

## Background

Osteoporosis occurring as a result of estrogen deficiency after the menopause is associated with a rapid increase in the risk of serious fractures [1]. Among such osteoporotic fractures, those of the hip present a major health problem with prolonged hospitalization, decreased quality of life, and increased risk of death [2]. It is estimated that the worldwide incidence of hip fractures will rise from 1.66 million in 1990 to 6.26 million by 2050 [3]. Therefore, to prevent hip fractures associated with minimal trauma in people with osteoporosis, effective treatments, which confer enhanced bone strength, particularly at the femoral neck, are needed.

Bisphosphonates inhibit bone resorption as they are selectively incorporated into osteoclasts and interfere with the resorptive action of osteoclasts [4]. Alendronate is a second generation bisphosphonate, and is widely used for postmenopausal, male, and glucocorticoid-induced osteoporosis. The effect of a single treatment of alendronate for postmenopausal osteoporosis to prevent femoral neck fracture has been shown in clinical [5,6], and animal studies [7,8].

Vitamin D_3 _is also given as a treatment for osteoporosis; evidence that vitamin D_3 _[1,25(OH)_2_D_3_] increases bone mineral density (BMD) and reduces hip fractures in postmenopausal osteoporosis has been reported [9], but other studies have shown no effect of the drug on bone mass [10,11]. Therefore, the effect of vitamin D_3 _for treatment of osteoporosis women remains controversial. However, in animal studies, vitamin D_3 _has been reported to prevent cortical and cancellous bone loss owing to estrogen deficiency by inhibiting bone resorption [12,13]. Vitamin D_3 _at higher doses shows a bone anabolic action by enhancing osteoblast activity [14].

A combination of two different drugs is believed to be a more effective treatment than a single treatment for osteoporosis; the combination of bisphosphonate and a bone anabolic drug has been used clinically [15,16]. Combined alendronate and calcitriol treatments have been reported to be more beneficial for BMD of the femoral neck than either alendronate or calcitriol alone in postmenopausal osteoporosis [15]. BMD is the most clinically relevant determinant of bone strength in human osteoporosis. However, there are no satisfactory clinical means to investigate the relationship between BMD and bone strength; therefore, the relationship has been investigated in ovariectomized rats as a model of postmenopausal osteoporosis [7,8,17-20]. To the best of our knowledge, no study has assessed whether the combination of alendronate and vitamin D_3 _enhances the mechanical strength of femoral necks, which clinically is a more interesting site at which to assess measurements.

Owing to the different mechanisms of action of these agents, our hypothesis was that the combination of alendronate and vitamin D_3 _would facilitate greater improvements in bone mass and strength at the femoral neck than either intervention alone. Therefore, we aimed to investigate the effect of combined treatment with alendronate and calcitriol on bone mass by assessing peripheral quantitative computed tomography (pQCT) and on fracture load at the femoral neck in ovariectomized rats, and comparing the bone mass parameters with the fracture load at the femoral neck.

## Methods

### Experimental design

This study protocol was approved by the Fukuoka University Animal Care and Use Committee. Thirty 11-week-old female Wistar rats, mean weight 277.9 (SD 13.4) g, were purchased from Seac Co. Ltd (Fukuoka, Japan) and acclimated to conditions for 1 week before the experiments. All rats were maintained in separate plastic cages under normal conditions (22–26°C; air humidity 55–60%; 12 h light/dark cycle). They had free access to food and water. At 12 weeks of age the rats were randomly divided into two groups, bilaterally ovariectomized (OVX: n = 24) and sham-operated (Sham-control: n = 6) under anaesthesia induced by intraperitoneal injection with sodium pentobarbital (40 mg/kg/ml, Dinabot Inc, Osaka, Japan). Twenty weeks after surgery, the OVX rats were further divided into four groups (n = 6 per group). The four OVX groups were treated for 12 weeks with daily doses of either saline alone, 0.1 mg/kg of alendronate (monosodium 4-amino-1-hydroxybutylidene-1, 1-diphosphonate trihydrate), 0.1 μg/kg of calcitriol [1,25(OH)_2_D_3_], or a treatment combining alendronate and calcitriol. Doses were delivered by mini-osmotic pumps (Alzet Pump, Alza Corporation, Palo Alto, CA) implanted subcutaneously and replaced every 4 weeks. Alendronate was dissolved in phosphate-buffered saline, and calcitriol was dissolved in propylene glycol immediately before implantation. The 0.1 mg/kg alendronate and 0.1 μg/kg calcitriol doses were chosen according to the results of earlier studies [21-23]. The sham-control group received saline alone by mini-osmotic pumps for 12 weeks. After 12 weeks of treatment, all animals were sacrificed with an overdose of pentobarbital and their bilateral femora excised.

### pQCT densitometry

The cross-sections of the left femoral necks were scanned using a pQCT system (XCT Research SA+, Software version 5.50e, Stratec Medizintechnik GmbH, Pforzhein, Germany). This system has a 50 kV/0.3 mA X-ray source. On a scout view of the femoral neck, three scan lines were manually placed so that the cross-sectional slice passed at every 0.2 mm through the mid-point and the proximal and distal ends of the longitudinal axis of the femoral neck. The scan time was 7.0 min and voxel size 0.08 × 0.08 × 0.46 mm. At the three points of the femoral neck, total bone mineral content (total BMC), total bone mineral density (total BMD), cortical BMC, cortical BMD, cancellous BMC, cancellous BMD, cortical bone thickness and total cross-sectional bone area (total bone area) were recorded using the pQCT software. The means of these values at the three points were then calculated. The coefficient of variation for the pQCT measurements was less than 5%.

### Mechanical testing

The mechanical strength of the femoral neck was measured by applying a vertical load to the femoral head using a Shimadzu EZ-1 pressure system (Shimadzu, Osaka, Japan). After the femora had been slowly thawed at room temperature, soft tissues were removed from the femora and the shafts of the femora cut at the mid-shaft of each femur. The distal femora were fixed with methylmethacrylate cement up to the lesser trochanter, maintaining a vertical position [17,18]. A vertical load from a brass cylinder was applied to the top of the femoral head. The cylinder was directed parallel to the axis of the femoral diaphysis and moved at a constant displacement speed of 5 mm/min until the femoral neck fractured. The fracture load was recorded at the peak force as Newton (N) at the point that the femoral neck fractured.

### Statistical analysis

The results for each group are expressed as means ± standard deviation (SD). The comparison between the sham-operation group (n = 6) and the OVX control group (n = 6) was done by unpaired t-tests. Differences between treated groups (n = 6 per group) were calculated by two-way factorial analysis of variance (ANOVA) test followed by Tukey's multiple comparison test as a post hoc test. Linear regression analysis was used to correlate bone mass parameters with bone strength. P values of < 0.05 were considered significant.

## Results

### pQCT densitometry

**Figure 1:**
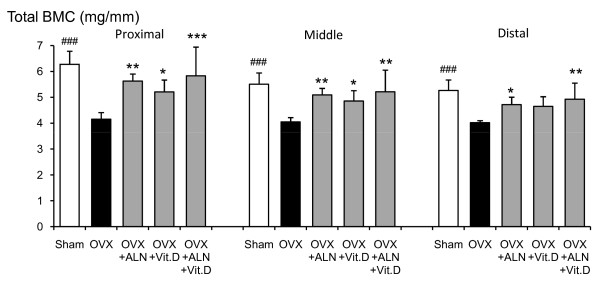
**Total BMC at the proximal, middle and distal parts of the femoral neck measured by pQCT densitometry**. Effects of alendronate (ALN), calcitriol (VitD), and combined alendronate and calcitriol treatments (ALN+VitD) on the pQCT densitometric parameters of total BMC at the 3 parts of the femoral neck in OVX rats after 12 weeks of treatment are shown. The data are presented as mean ± SD (n = 6 per group). ^### ^P < 0.001 as compared with corresponding values in saline-treated OVX (unpaired t-test). * P < 0.05, ** P < 0.01, *** P < 0.001 as compared with corresponding values in saline-treated OVX (Tukey's multiple comparison test). Total BMC at the 3 parts of the femoral neck was significantly lower in the saline-treated OVX compared with the sham group. Alendronate treatment, with or without calcitriol, induced a significant improvement in all total BMC.

**Figure 2:**
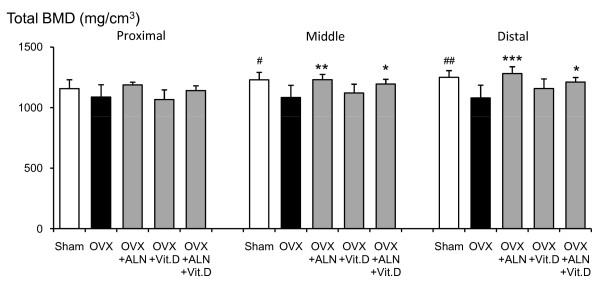
**Total BMD at the proximal, middle and distal parts of the femoral neck measured by pQCT densitometry**. Effects of alendronate (ALN), calcitriol (VitD), and combined alendronate and calcitriol treatments (ALN+VitD) on the pQCT densitometric parameters of total BMD at the 3 parts of the femoral neck in OVX rats after 12 weeks of treatment are shown. The data are presented as mean ± SD (n = 6 per group). ^# ^P < 0.05, ^## ^P < 0.01 as compared with corresponding values in saline-treated OVX (unpaired t-test). * P < 0.05, ** P < 0.01, *** P < 0.001 as compared with corresponding values in saline-treated OVX (Tukey's multiple comparison test). In the middle and distal parts of the femoral neck, total BMD was significantly lower in the saline-treated OVX compared with the sham group. In total BMD of these parts, alendronate treatment, with or without calcitriol, showed a significant improvement.

**Figure 3:**
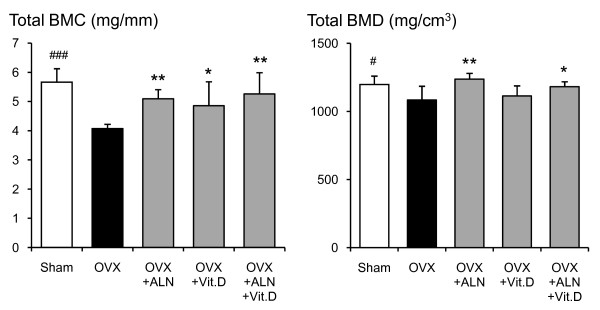
**Total BMC and BMD at the femoral neck measured by pQCT densitometry**. Effects of alendronate (ALN), calcitriol (VitD), and combined alendronate and calcitriol treatments (ALN+VitD) on the pQCT densitometric parameters of total BMC and BMD at the femoral neck in OVX rats after 12 weeks of treatment are shown. The data are presented as mean ± SD (n = 6 per group). ^# ^P < 0.05, ^### ^P < 0.001 as compared with corresponding values in saline-treated OVX (unpaired t-test). * P < 0.05, ** P < 0.01 as compared with corresponding values in saline-treated OVX (Tukey's multiple comparison test). Total BMC and BMD were significantly lower in the saline-treated OVX compared with the sham group. Alendronate treatment, with or without calcitriol, induced a significant improvement in both total BMC and BMD.

**Figure 4:**
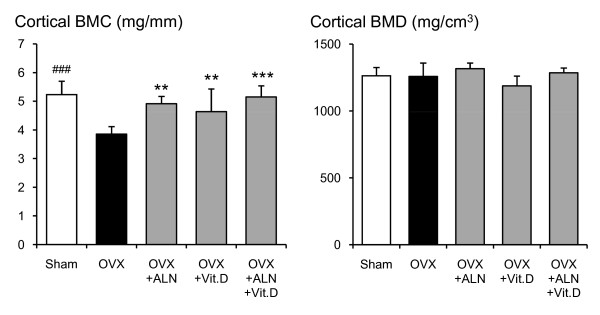
**Cortical BMC and BMD at the femoral neck measured by pQCT densitometry**. Effects of alendronate (ALN), calcitriol (VitD), and combined alendronate and calcitriol treatments (ALN+VitD) on the pQCT densitometric parameters of cortical BMC and BMD at the femoral neck in OVX rats after 12 weeks of treatment are shown. The data are presented as mean ± SD (n = 6 per group). ^### ^P < 0.001 as compared with corresponding values in saline-treated OVX (unpaired t-test). * P < 0.05, ** P < 0.01, *** P < 0.001 as compared with corresponding values in saline-treated OVX (Tukey's multiple comparison test). Cortical BMC was significantly lower in the saline-treated OVX than in the sham group. Cortical BMC in OVX rats was significantly improved by alendronate, calcitriol single treatment, and the combined treatment.

**Figure 5:**
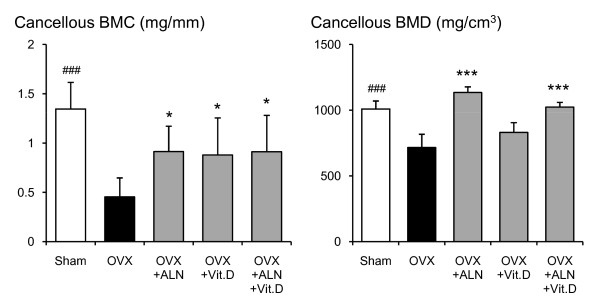
**Cancellous BMC and BMD at the femoral neck measured by pQCT densitometry**. Effects of alendronate (ALN), calcitriol (VitD), and combined alendronate and calcitriol treatments (ALN+VitD) on the pQCT densitometric parameters of cancellous BMC and BMD at the femoral neck in OVX rats after 12 weeks of treatment are shown. The data are presented as mean ± SD (n = 6 per group). ^### ^P < 0.001 as compared with corresponding values in saline-treated OVX (unpaired t-test). * P < 0.05, *** P < 0.001 as compared with corresponding values in saline-treated OVX (Tukey's multiple comparison test). OVX significantly reduced cancellous BMC and BMD. In alendronate single and the combined treatment group, a significant increment in both cancellous BMC and BMD was found. Calcitriol single treatment significantly improved cancellous BMC only.

**Figure 6:**
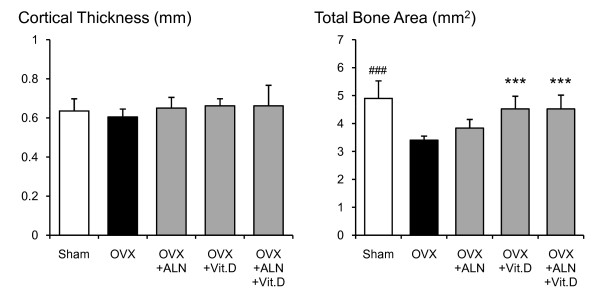
**Cortical thickness and total bone area at the femoral neck measured by pQCT densitometry**. Effects of alendronate (ALN), calcitriol (VitD), and combined alendronate and calcitriol treatments (ALN+VitD) on the pQCT densitometric parameters of cortical thickness and total bone area at the femoral neck in OVX rats after 12 weeks of treatment are shown. The data are presented as mean ± SD (n = 6 per group). ^### ^P < 0.001 as compared with corresponding values in saline-treated OVX (unpaired t-test). *** P < 0.001 as compared with corresponding values in saline-treated OVX (Tukey's multiple comparison test). Total bone area was significantly decreased by OVX. Calcitriol single and the combined treatment induced a significant improvement in total bone area.

Total BMC at the proximal, middle and distal parts of the femoral neck in saline-treated OVX group was significantly lower than those in the sham-control group (p < 0.001, p < 0.001, and p < 0.001, respectively) (Fig. 1). Alendronate and calcitriol alone, and in combination significantly improved total BMC in the proximal (p = 0.003, p = 0.041, and p < 0.001, respectively) and middle parts (p = 0.007, p = 0.044, and p = 0.003, respectively) of the femoral neck. The total BMC at the distal parts with alendronate alone or in combination with calcitriol was significantly higher than that in the saline-treated OVX group (p = 0.028 and p = 0.004, respectively). In the middle and distal parts, the total BMD of the saline-treated OVX group was lower than that of the sham-control group (p = 0.013 and p = 0.006, respectively) (Fig. 2). The total BMD at the middle and distal parts was significantly higher with alendronate alone than in the saline-treated OVX group (p = 0.007 and p < 0001, respectively). Total BMD at the distal parts with combined treatment was greater than that in the saline-treated OVX group (p = 0.031). The means of total BMC, total BMD, cortical BMC, cortical BMD, cancellous BMD, cancellous BMC, cortical bone thickness and total bone area at the three parts are shown in Figures 3, 4, 5, 6.

In the saline-treated OVX group, a statistically significant reduction of total BMC by 28.1% (p < 0.001), total BMD by 9.5% (p = 0.016), cortical BMC by 26.3% (p < 0.001), cancellous BMC by 66.3% (p < 0.001), cancellous BMD by 29.0% (p < 0.001), and total bone area by 30.4% (p < 0.001) were shown when compared with the sham-control group (Figs. 3, 4, 5, 6). Alendronate and calcitriol alone, and in combination significantly improved total BMC (p = 0.009, p = 0.046, and p = 0.006; respectively; Fig. 3), cortical BMC (p = 0.005, p = 0.009, and p < 0.001, respectively; Fig. 4), and cancellous BMC (p = 0.033, p = 0.045, and p = 0.033, respectively; Fig. 5) in OVX rats compared with the saline-treated OVX group. Additionally, with both alendronate alone and in combination with calcitriol, total BMD (p = 0.005 and p = 0.047, respectively; Fig. 3) and cancellous BMD (p < 0.001 and p < 0.001, respectively; Fig.5) were significantly greater than in the saline-treated OVX group. Calcitriol alone and in combination with alendronate achieved significantly higher total bone area values in OVX rats compared with the saline-treated OVX group (p < 0.001 and p < 0.001, respectively; Fig. 6).

### Mechanical testing

**Figure 7:**
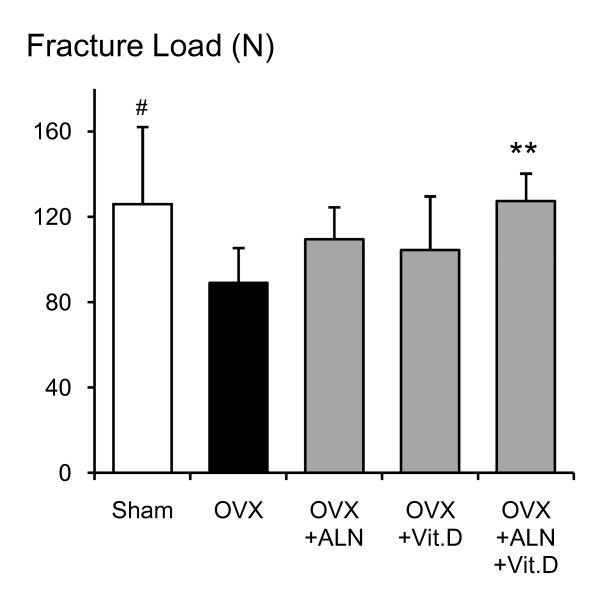
**Mechanical strength of the femoral neck**. Mechanical results of femoral neck fractures in sham control rats, and OVX rats that received saline, alendronate (ALN), calcitriol (VitD), or combined alendronate and calcitriol treatments (ALN+VitD) for 12 weeks. The data are presented as mean ± SD (n = 6 per group). ^# ^P < 0.05 as compared with corresponding values in saline-treated OVX (unpaired t-test).* P < 0.05 as compared with corresponding values in saline-treated OVX (Tukey's multiple comparison test). Bone strength of the femoral neck was significantly lower in the saline-treated OVX compared with the sham group. The bone strength in OVX rats was significantly improved by the combined alendronate and calcitriol treatment but not by alendronate or calcitriol single treatment.

The average maximum fracture loading to the femoral necks was 29.3% lower in the saline-treated OVX group compared with the sham-control group (p = 0.046) (Fig. 7). Femoral neck strength in all treated OVX groups was higher than that in the saline-treated OVX group, but only the combined treatment showed a significant difference compared with the saline-treated OVX group (p = 0.007). All femoral neck fractures were observed to be midcervical.

### Relationship between bone mass parameters and fracture load at the femoral neck

In all groups, total BMC, cortical BMC and total bone area were found to be correlated with fracture load at the femoral neck, with total BMC showing the highest; in contrast, total BMD and fracture load were not significantly correlated (Additional file 1, Fig. 8). Additionally, in the alendronate and calcitriol alone or in combination groups, total BMC and fracture load were significantly correlated (Additional file 1).

**Figure 8:**
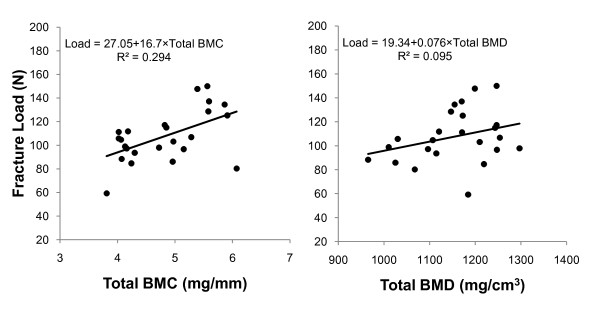
**Relationship between bone mass and fracture load at the femoral neck**. A relationship was found between total BMC and fracture load at the femoral neck, but no relationship between total BMD and fracture load at the femoral neck was observed. The values of the correlation coefficient (*r*) and correlation significance (*p*) are described in Additional file 1.

## Discussion

Our study revealed that combination therapy with alendronate and calcitriol significantly improved bone mass and femoral neck strength in OVX rats. The results indicated that total BMC values had the strongest correlation with the mechanical strength of the femoral neck.

Several studies [7,8] have demonstrated the effect of a single treatment of alendronate on the mechanical strength of the femoral neck in OVX rats. In the first study, starting alendronate immediately after OVX at dose of 0.04 mg/kg/day and 1.0 mg/kg/day significantly increased the strength of the femoral neck in OVX rats after 8 weeks of treatment [7]. However, the effects of alendronate on femoral neck strength in these OVX rats were not dose dependent. The second study [8] showed that the femoral neck strength of rats receiving alendronate at a dose of 3 mg/kg/day was significantly greater than that of OVX control rats, even though alendronate was administered to OVX rats for 4 weeks and started on the second day after OVX. In the present study, the dose of 0.1 mg/kg/day of alendronate increased the maximum fracture loading at the femoral neck in OVX rats, but this was not significant compared with the saline-treated OVX rats. The difference between the two above reports and our results may be due to differences in the period of time from ovariectomy to initiating treatment. It is possible that this period of time may also be partly responsible for differences in results with vitamin D_3 _treatment between postmenopausal women with osteoporosis [10,11] and ovariectomized rats [12,13]. Since osteoporosis associated with estrogen deficiency is a silent disease, when patients start treatment with various drugs, osteoporosis is already well established; therefore, in the present study all treatments were started at 20 weeks after ovariectomy.

Our OVX rats had significantly decreased total BMC, total BMD, cortical BMC, cancellous BMC, cancellous BMD, and total bone area at the femoral neck compared with sham-controls. The combination therapy with alendronate and calcitriol reverted these levels towards those observed in the sham-operated rats. However, these findings, excluding that for total bone area, were also observed with alendronate alone. Therefore, no synergistic effects of alendronate and calcitriol were observed in terms of bone mass in OVX rats.

In terms of the mechanical strength of the femoral neck, the present study showed no difference between alendronate alone or in combination with calcitriol, but only combination treatment of alendronate and calcitriol for 12 weeks significantly improved bone fragility owing to ovariectomy compared with the saline-treated OVX rats. For this reason, we also believe it is of interest that OVX rats treated with the combined treatment showed enhanced total BMC, cortical BMC, and total bone area, which are all correlated with femoral neck strength. Among the three parameters in bone mass, total BMC and cortical BMC were increased by alendronate, and total bone area was improved by calcitriol. Therefore, a combined treatment that reflects differences in functions of the two agents on bone mass, might significantly improve femoral neck strength.

Even though clinical studies have reported correlations between BMD and the incidence of femoral neck fracture in osteoporosis patients [5], our results found no correlation between BMD and femoral neck strength in rats. In fact, in our study, femoral neck strength was only correlated total BMC, cortical BMC, and total bone area. Our findings are in agreement with several other reports [24,25]. In healthy rats, femoral neck strength was shown to be correlated only with BMC and not BMD [24]. Meanwhile, in gastrectomized osteopenic rats, BMD was not correlated with femoral neck strength [25]. Furthermore, in another study, in which OVX rats were treat with human insulin-like growth factor-I (IGF-I) alone or in combination with pamidronate, although BMD and BMC were correlated with femoral neck strength, the association was stronger for BMC than for BMD [17]. In addition, cortical bone properties are of great interest in osteoporosis, since it is widely speculated that cortical bone quality does affect fracture risk. In this study, we measured cortical BMC, BMD and cortical thickness as markers of cortical bone quality. Of these, cortical BMC was positively correlated with the femoral neck strength in OVX rats. However, since the femoral neck strength is also affected by other parameters such as external diameter of the femoral neck, hip axis length [26], cortical porosis, mean degree of mineralization, and osteocytes, it may be difficult to evaluate the determinants of neck strength.

We note several limitations of our study. First, although 12-week-old female rats with growing bones were used in the present study, a baseline control group was not included for comparison. However, as the treatments were started 20 weeks after ovariectomy and lasted 12 weeks, the rats were 8 months old (32 weeks) when treatment started, and 11 months old when bone mass and strength were investigated. Second, the modest number of rats means that the power of the study to demonstrate statistically significant differences was relatively low. With more rats, any synergistic effect of combination alendronate and calcitriol therapy on bone mass and strength might become evident. Finally, we did not examine bone strength of the femoral neck in a configuration simulating a fall to the lateral side. The fall configuration is clinically more relevant because most osteoporotic hip fractures are associated with a fall [27]. In the fall loading configuration, it is important to consider anteversion of the femoral neck. However, this has yet to be clarified in rats. Therefore, we investigated the fracture load at the femoral neck in a direction parallel to the femoral shaft axis; however, the axial loading may influence the fracture types owing to different internal stress distributions. Although approximately half of osteoporotic hip fractures are intertrochanteric in human [28], all fractures in the present study were found in midcervical region.

Clinical studies have shown that vitamin D_3 _is effective in increasing BMD and reducing hip fractures in postmenoposal osteoporosis [9]; however, others have failed to reproduce the same results [10]. On the other hand, in animal studies using OVX rats, vitamin D_3 _treatment prevented bone loss occurring as a result of estrogen deficiency [12,13]. Since the bone remodeling period in OVX rats is shorter than that in humans [29], the reason for the discrepancy between OVX rats and humans is may be related to enhanced remodeling activity in the rat. In addition, the discrepancy may be due to a genetic difference in the sensitivity to vitamin D between rats and humans [30].

In summary, the present study showed that combination therapy with alendronate and calcitriol significantly restored cortical and cancellous bone loss that was due to estrogen deficiency in OVX rats. Although no synergistic effects of the two agents were found in terms of bone mass at the femoral neck, the combined treatment does reflect the effects of the two agents. On mechanical testing, our results demonstrated that the combined treatment significantly improved bone fragility of the femoral neck in osteopenic conditions.

## Competing interests

The authors declare that they have no competing interests.

## Authors' contributions

YN and MN designed the research. YN, AF and SA did the experiment and analyzed the data. YN wrote the draft manuscript and MN, KH and AF revised the draft manuscript.

## Supplementary Material

Additional file 1 **Relation between bone mass parameters and fracture load at the femoral neck**. In all groups, there were significant correlations between the fracture load and total BMC, cortical BMC or total bone area at the femoral neck. In all groups, the alendronate-treated groups (ALN and ALN + Vit.D), the calcitriol-treated groups (Vit.D and ALN + Vit.D) and combined treatment group, there were significant correlation between the fracture load and total BMC in the femoral neck. Click here for file
