# Supplementary material for: Effect of combined treatment with alendronate and calcitriol on femoral neck strength in osteopenic rats
Source: J Orthop Surg Res. 2008 Dec 17;3:51. doi: 10.1186/1749-799X-3-51 (PMC2631529; doi:10.1186/1749-799X-3-51)
Supplement: Additional file 1 — Relation between bone mass parameters and fracture load at the femoral neck. In all groups, there were significant correlations between the fracture load and total BMC, cortical BMC or total bone area at the femoral neck. In all groups, the alendronate-treated groups (ALN and ALN + Vit.D), the calcitriol-treated groups (Vit.D and ALN + Vit.D) and combined treatment group, there were significant correlation between the fracture load and total BMC in the femoral neck. [file 1749-799X-3-51-S1.pdf]

Relation between bone mass parameters and fracture load at the femoral neck

Parameters	All groups		Alendronate-treated groups (ALN and ALN + Vit.D)		Vitamin D_3-treated groups (Vit.D and ALN + Vit.D)		Combined treatment group (ALN + Vit.D)	
	Correlation coefficient (*r*)	Significance (*p*)	Correlation coefficient (*r*)	Significance (*p*)	Correlation coefficient (*r*)	Significance (*p*)	Correlation coefficient (*r*)	Significance (*p*)
Total BMC	0.509	0.004	0.616	0.033	0.596	0.043	0.825	0.043
Total BMD	0.336	0.070	0.554	0.061	0.510	0.086	0.210	0.704
Cortical BMC	0.492	0.006	0.665	0.018	0.588	0.048	0.743	0.091
Cortical BMD	0.308	0.098	0.550	0.064	0.492	0.092	0.183	0.729
Cancellous BMC	0.351	0.058	0.393	0.207	0.171	0.595	0.421	0.405
Cancellous BMD	0.259	0.166	0.436	0.110	0.236	0.459	0.758	0.081
Cortical bone thickness	0.063	0.740	0.041	0.899	0.024	0.940	0.050	0.925
Total bone area	0.487	0.006	0.691	0.013	0.610	0.039	0.598	0.210
